# Data Field Modeling and Spectral-Spatial Feature Fusion for Hyperspectral Data Classification

**DOI:** 10.3390/s16122146

**Published:** 2016-12-16

**Authors:** Da Liu, Jianxun Li

**Affiliations:** School of Electronic, Information and Electrical Engineering, Shanghai Jiao Tong University, Shanghai 200240, China; lijx@sjtu.edu.cn

**Keywords:** feature fusion, data field theory, hyperspectral data, mathematical morphology, spectral-spatial classification

## Abstract

Classification is a significant subject in hyperspectral remote sensing image processing. This study proposes a spectral-spatial feature fusion algorithm for the classification of hyperspectral images (HSI). Unlike existing spectral-spatial classification methods, the influences and interactions of the surroundings on each measured pixel were taken into consideration in this paper. Data field theory was employed as the mathematical realization of the field theory concept in physics, and both the spectral and spatial domains of HSI were considered as data fields. Therefore, the inherent dependency of interacting pixels was modeled. Using data field modeling, spatial and spectral features were transformed into a unified radiation form and further fused into a new feature by using a linear model. In contrast to the current spectral-spatial classification methods, which usually simply stack spectral and spatial features together, the proposed method builds the inner connection between the spectral and spatial features, and explores the hidden information that contributed to classification. Therefore, new information is included for classification. The final classification result was obtained using a random forest (RF) classifier. The proposed method was tested with the University of Pavia and Indian Pines, two well-known standard hyperspectral datasets. The experimental results demonstrate that the proposed method has higher classification accuracies than those obtained by the traditional approaches.

## 1. Introduction

With the development of imaging instruments in the past few years, hyperspectral data processing has become increasingly more important in many fields [1,2,3,4,5]. As a data tool with high spectral resolution, hyperspectral sensors usually utilized hundreds of spectral channels to describe spectral signatures. Generally, the primary purpose of hyperspectral images (HSI) processing is to analyze and recognize spectral data acquired by hyperspectral sensors. It is established that, different materials have distinct reflectance spectral signatures. Thus, reflectance spectra are always used for material recognition and image analysis [6].

However, while the high dimensionality of HSI supports accurate descriptions for spectral signatures, they lead to some theoretical and practical problems, particularly the curse of dimensionality problem. In classification problems, classification accuracies are not positively correlated to the dimensionality of input data. Usually, classification is most accurate with a particular feature number, as has been demonstrated in References [7,8,9]. Hence, feature extraction and dimensionality reduction techniques are important and indispensable in high-dimensional data classification and analysis. Based on known information, feature extraction (FE) techniques are generally categorized into unsupervised and supervised methods. Unsupervised FE techniques, e.g., principle component analysis (PCA) [10], are always used for data description and representation. Supervised FE focuses on reducing the dimensionality of data to achieve better classification performance and avoid Hughes phenomena [7]. Many supervised feature extraction algorithms have been proposed and widely used in hyperspectral image processing, such as the discriminant analysis feature extraction (DAFE) algorithm [11], the decision boundary feature extraction (DBFE) approach [12], and the nonparametric weighted feature extraction (NWFE) method [13], etc.

Conventionally, HSI are treated by classifiers as spectral data cubes and a set of spectral measurements without spatial structure [14]. Hence, the spatial structure features in HSI are discarded. However, with the development of sensors, HSI usually provides both detailed spatial structural and spectral information. Crisp and adaptive neighborhood systems are commonly used to characterize spatial structural features [15]. The crisp system generally analyzes the spatial structure based on a tough neighborhood. The crisp system is widely used in spatial information extraction. However, it has the following limitations: (1) the classifier effectiveness may be influenced by the predefined neighborhood system without enough samples; and (2) a large neighborhood system usually results in computation problems [16]. For this reason, adaptive neighborhood systems are also taken into account. Based on the morphology theory [17], which has been widely used in image processing, a set of methods for spatial information extraction using adaptive neighborhood systems [18,19,20,21,22,23,24] have been proposed.

Morphological profiles (MPs) [18] have demonstrated their usefulness in spatial structure description. The sizes of different structures in an image can be determined by using geodesic opening/closing through reconstruction [19,20]. For any given size of a structuring element (SE), the structures that are smaller than the SE are removed, while larger structures are preserved. The spatial information of the image is extracted by applying such operators with an SE range of different sizes. This concept is usually called granulometry [21]. The attribute profiles (APs) technique [22] is a further development of MPs based on attribute filters, which allow for the modeling of geometrical characteristics. Compared with MPs, APs allow more precise modeling of spatial information. This is because an input image can be processed based on multiple attributes, by which different aspects of spatial structures can be described with great flexibility. When dealing with vectorial images, typically HSI, the application of morphological filters has been extended based on the concept of the vectorial image profile. Extended morphological profiles (EMPs) [23] and extended attribute profiles (EAPs) [21] were proposed to extract the spectral and spatial features of the hyperspectral data. In References [21,23], PCA was first implanted in original hyperspectral data, and the first principle components that contained particular cumulative variance were selected as the baseline images. Then MPs and APs were performed on all the selected PCs. EMP and EAP were composed by these MPs and APs, respectively. In later studies, an extended multi-attribute profile (EMAP) was proposed in References [15,24]. EMAP, which utilizes multi-attributes, is a more advanced version of EAP. Additionally, Reference [25] proposed a supervised feature selection approach in attribute profiles on the basis of a genetic algorithm (GA). By introducing the GA technique, the EMAPs with the highest importance are preserved for classification. In References [15,25], supervised FE techniques were used to create better profiles and extract more discriminate spatial features. In Reference [26], a state-of-the-art hyperspectral classification based on sparse representation and EMAPs was proposed. Based on the fact that the extracted EMAPs with high dimensionality should have particular class-dependent manifold structures, this classification approach exploits the inherent characteristics of EMAPs embedded in high-dimensional feature space. This method, called SUnSAL in Reference [26], combines the benefits of sparse representation and the rich spatial structural information obtained by EMAPs.

In order to consider both the spectral features and spatial features, spectral-spatial classifiers have become increasingly important in HSI classification. A few studies, such as References [27,28], have proposed several spectral-spatial FE methods based on supervised FE techniques and morphological filters for HSI classification. In References [27,28], the spectral and spatial features were extracted using supervised FE approaches and morphological filters, and then the extracted spectral features and spatial features were fused via vector stacking. Thus, both spectral and spatial information were utilized in classification. Reference [29] extracted the local image structures by employing local binary patterns (LBP). LBP features were extracted on all selected spectral bands. Next, the local image patterns and spectral features were fused both at the feature and decision level for classification. In Reference [30], a spectral-spatial method using multi-hypothesis (MH) prediction for noise-robust HSI classification was proposed. By using a weighted regularization, the MH prediction finds the best linear hypothesis combination and achieves spectral-spatial classification. Inspired by the deep leaning idea, a deep feature extraction algorithm based on convolution neural networks was presented in Reference [31]. However, it is important to note that the inner relationship between spatial and spectral features has received little attention. In order to further improve the classification accuracy, new information must be introduced and explored, particularly the information hidden in the relationship between the spectral and spatial information. References [32,33,34] have demonstrated that spatial neighbors always contribute to the measured signal through adjacency effects. Hence, spectral and spatial features are not independent due to data interaction in HSI.

In this study, a supervised spectral-spatial classification algorithm based on data field theory is proposed. This algorithm improves classification accuracy by further processing the extracted spectral and spatial information. Unlike the current classification approaches, the proposed method aims to further improve classification performance by exploring the inner relationship between spectral and spatial information. The main motivation of the proposed method is that data influences and interactions should be taken into consideration, which is often neglected in spatial-spectral classification tasks. By considering the mutual influences and interactions between pixels, we attempt to build the connection between the spectral and spatial domains. So, more useful information hidden in the relationship between spectral and spatial information, or, for simplicity, the adjacency effects, can be explored and included for classification. In our study, spectral information was extracted by supervised FE techniques and spatial information was generated by EMAP, as performed in Reference [27]. Next, data field modeling was applied to both the spectral and spatial domains. Based on data field modeling, the spectral and spatial information are unified. So, the unified radiation features containing both spectral and spatial information can be fused into a new radiation feature by using a linear model. Another advantage of data field modeling in both spectral and spatial domains is that the problem of the extracted spectral and spatial features having different scales can be avoided. An random forest (RF) classifier provides a final classification map [35]. The novelty of the proposed algorithm lies in its use of data field theory to explore the relationship between the spatial and spectral information. To measure the efficacy of the presented method, we tested it by using two standard hyperspectral datasets. In the remainder of this paper, Section 2 covers the detailed presentation of the proposed algorithm. Section 3 presents a series of experiments with two standard HSI test datasets. In Section 3, the experimental results of different test cases are analyzed and key parameters used in the proposed method are discussed. The advantages of the proposed approach and proposed subjects for future investigation are drawn in Section 4, followed by the conclusions in Section 5.

## 2. The Proposed Method 

### 2.1. Data Field Modeling

Data fields are the mathematical expression of field theory in physics. Data fields establish models in which data can be seen as a whole by studying the interactions of data. To describing the relationship between data, data are treated as radiation sources within the data field. Thus, the radiation effect can be used to mathematically describe the data interaction. Employing this approach, the property of a vector point is determined not only by its location in the data space, but also by the other surrounding data in the data field owing to the radiation effect. In this paper, both the spectral and spatial domains of an HSI are considered as data fields. Thus, the recognition and identification of a pixel in a HSI do not depend only on its position in the spectral space—on its spectral signature for simplicity—but also take into account its interactions with the other pixels in the HSI.

In this paper, we define the radiation intensity as a function that depends on a distance measurement. The function is called the radiation function, and is mathematically expressed as:

(1) $$E(\rho ,d)=E_{0}\exp\{-\rho \cdot d^{2}\}$$

where d denotes the distance to the radiation source, *E* is the radiation intensity at d, ρ is a radiation factor and $E_{0}$ indicates the initial energy. Both Mahalanobis and Euclidean distances are employed as the distance measurements in this paper. We term the Mahalanobis distance $d_{M}$ and the Euclidean distance $d_{E}$. Apparently, while d is small, the points in a data pair interact with each other intensively. In contrast, the $e^{-\rho d^{2}}$ term tends toward zero and the interaction is negligible when d is large. The radiation function allows us to establish the connections between the data in data fields and to describe the interactions between the data pairs as radiation intensities.

Suppose $x={\left[x_{\varphi },x_{\omega }\right]}^{T}$ is a feature vector that corresponds to a pixel in HSI; here, $x_{\varphi }$ represents the spectral feature extracted by supervised FE techniques, and $x_{\omega }$ denotes the spatial structural feature. In the following description, the symbols related to spectral space are denoted by suffix φ and those related to spatial space are denoted by ω. Thus, a pixel in HSI corresponds to a feature vector $x_{\varphi }$ in the spectral feature space $R_{\varphi }$, and a feature vector $x_{\omega }$ in the spatial feature space $R_{\omega }$. In this paper, both $R_{\varphi }$ and $R_{\omega }$ are considered data fields. Thus, a data point receives radiations in both $R_{\varphi }$ and $R_{\omega }$. Furthermore, we suppose that all the data have a unit initial radiation energy, i.e., $E_{0}=1$, when data field modeling in both the spectral and spatial domains.

Suppose a training sample set ${\left\{(x_{i},u_{i})\right\}}_{i=1}^{N}$, where $x_{i}={\left[x_{i,\varphi },x_{i,\omega }\right]}^{T}$ denotes an input pattern, $u_{i}\in \{1,...,L\}$ denotes its class label, and N and L are the numbers of the training samples and classes, respectively. For training sample $\left(x_{i},l\right)$, according to the label $u_{i}=l$, two subsets of the training set are defined. The first subset contains all the training samples that have the same label as $x_{i}$, and we term this subset the Same Class Subset. The other subset contains all the training samples with class labels different from $x_{i}$, and is called the Different Class Subset. We suppose that a given training sample $x_{i}$ receives radiation from its *k*-nearest training samples in every class. For example, ${\left\{(s_{j},v_{j})\right\}}_{j=1}^{k\times L}$ denotes the set of nearest neighbors (NNs) with respect to $\left(x_{i},l\right)$, and $s_{j}$ is the *j*th nearest neighbor (NN) with a class label $v_{j}$, where j=1,...,k×L, $v_{j}=1,...,L$. We have $x_{i}={\left[x_{i,\varphi },x_{i,\omega }\right]}^{T}$ and $s_{j}={\left[s_{j,\varphi },s_{j,\omega }\right]}^{T}$. Then, radiations from $s_{j}$ to $x_{i}$ in $R_{\varphi }$ and $R_{\omega }$ are respectively defined as:

(2) $$\left\{ \begin{array}{l} e_{j,\omega }=\exp\{-\rho_{\omega }^{l}d_{M}{\left(x_{i,\omega },s_{j,\omega }\right)}^{2}\}\\ e_{j,\varphi }=\exp\{-\rho_{\varphi }^{l}d_{M}{\left(x_{i,\varphi },s_{j,\varphi }\right)}^{2}\} \end{array}\right.$$

Here, $\rho_{\varphi }^{l}$ and $\rho_{\omega }^{l}$ are the radiation factors of $\left(x_{i},l\right)$ in the spectral domain data field and spatial domain data field, respectively, and $d_{M}(\cdot )$ denotes the Mahalanobis distance. It should be noted that we used different radiation factors in different spaces and classes. The radiation factors can be determined by the training samples, and will be discussed in the following section. Consequently, $x_{i}$ is projected as:

(3) $$x_{i}\mapsto x_{i}^{e}={\left[e_{1,\varphi }^{1},...,e_{N_{c},\varphi }^{k},e_{1,\omega }^{1},...,e_{N_{c},\omega }^{k}\right]}^{T}\in {\mathbb{R}}^{m}$$

where ${\mathbb{R}}^{m}$ denotes an *m*-dimensional space, $m=2\times N_{k}$, and $N_{k}=k\times N_{c}$ is the number of the NNs in all classes. We term $x_{i}^{e}$ the data field radiation feature (DFRF).

In this paper, we define the total radiation as a weighted addition of the radiations in both $R_{\varphi }$ and $R_{\omega }$, i.e., the total radiation $x_{i}$ received from $s_{j}$ can be defined as:

(4) $$e_{j}=\alpha e_{j,\varphi }+(1-\alpha )e_{j,\omega },\;0<\alpha <1$$

where 0<α<1 is a weight coefficient. In Equation (4), the first term on the right indicates the spectral information of the input pattern describing the radiation received in the spectral feature space. The second term represents the radiation in the spatial feature space that can be seen as the spatial information. The weight coefficient α is used to describe the inner connection between spectral and spatial features. Hence, Equation (4) contains the spatial features, spectral features and the spectral-spatial information relationship. The left term in Equation (4) fuses the radiation in the spectral and spatial spaces into a total radiation feature. In essence, the data modeling operation is a feature-unifying and fusion procedure. Following the data field modeling approach presented here, the data radiation interactions are built in both the spatial and spectral domains. Hence, the spectral and spatial feature domains are unified and correlated. Consequently, the spatial and spectral information are unified and fused through the data field modeling, and $x_{i}$ is transferred to $y_{i}$ which is termed as the fused data field radiation feature (FDFRF):

(5) $$x_{i}\mapsto y_{i}={\left[e_{1}^{1},...,e_{1}^{k},...,e_{N_{c}}^{1},...,e_{N_{c}}^{k}\right]}^{T}\in {\mathbb{R}}^{N_{k}}$$

### 2.2. Weight Coefficient Training

The weight coefficient α describes the inner connection between spectral and spatial features. We discuss the method of determining the value of the coefficient in this section. For a given training sample $\left(x_{i},u_{i}\right)$, the $k_{1}$-nearest-neighbors ($k_{1}$-NN) in its Same Class Subset are selected and denoted by $x_{i_{1}},\ldots ,x_{i_{k_{1}}}$, while $k_{2}$-NN in the Different Class Subset are selected and denoted by $x_{i^{1}},\ldots ,x_{i^{k_{2}}}$. Then, the data patch of $x_{i}$ can be built as:

(6) $$X_{i}=[x_{i},x_{i_{1}},...,x_{i_{k_{1}}},x_{i^{1}},...,x_{i^{k_{2}}}]$$

The corresponding DFRF and FDFRF are, respectively:

(7) $$X_{i}^{e}=[x_{i}^{e},x_{i_{1}}^{e},...,x_{i_{k_{1}}}^{e},x_{i^{1}}^{e},...,x_{i^{k_{2}}}^{e}]\;\mathrm{and}\;Y_{i}=[y_{i},y_{i_{1}},...,y_{i_{k_{1}}},y_{i^{1}},...,y_{i^{k_{2}}}]$$

It is easy to derive that $Y_{i}=\left(\alpha A+B\right)X_{i}^{e}$, where, A=[I|−I], B=[0|I] and I is an identity matrix. For the FDFRFs in each patch, we want the distances between $y_{i}$ and $y_{i_{1}},\ldots ,y_{i_{k_{1}}}$ to be as small as possible. Meanwhile, the distances between $y_{i}$ and $y_{i^{1}},\ldots ,y_{i^{k_{2}}}$ are as large as possible. So, we have:

(8) $$\arg\underset{y_{i}}{\min}\;\left(\sum_{j=1}^{k_{1}}d_{E}{\left(y_{i},y_{i^{j}}\right)}^{2}-\beta \sum_{p=1}^{k_{2}}d_{E}{\left(y_{i},y_{i^{p}}\right)}^{2}\right)$$

where β∈[0,1] is a scaling factor, and $d_{E}(\cdot )$ represents the Euclidean distance. Local information is introduced to train the weight coefficient. The local information specifies the subspaces in which the boundary regions are embedded and deemphasizes those samples far from the boundaries. We define the $\lambda_{i}$ as:

(9) $$\lambda_{i}=d_{E}(x_{i}^{e},M_{i}^{e})/\left[d_{E}(x_{i}^{e},M_{i}^{e})+\underset{1\leq j\leq k_{2}}{\min}d_{E}(x_{i}^{e},x_{i^{j}}^{e})\right]$$

where $\lambda_{i}\in [0,1]$ describes the local information, $M_{i}^{e}$ is the center of $x_{i_{1}}^{e}\ldots ,x_{i_{k_{1}}}^{e}$, and $\underset{1\leq j\leq k_{2}}{\min}d_{E}\left(x_{i}^{e},x_{i^{j}}^{e}\right)$ denotes the minimum distance from $x_{i}^{e}$ to $x_{i^{1}}^{e},\ldots ,x_{i^{k_{2}}}^{e}$. Then Equation (8) can be changed into:

(10) $$\arg\underset{y_{i}}{\min}\;\lambda_{i}\left(\sum_{j=1}^{k_{1}}d_{E}{\left(y_{i},y_{i^{j}}\right)}^{2}-\beta \sum_{p=1}^{k_{2}}d_{E}{\left(y_{i},y_{i^{p}}\right)}^{2}\right)$$

Furthermore, we define a coefficients vector and patch matrix:

(11) $$\omega_{i}={\left[\underset{k_{1}}{\underset{︸}{1,...,1}},\underset{k_{2}}{\underset{︸}{-\beta ,...,-\beta }}\right]}^{T}\;\mathrm{and}\;L_{i}=\left[\begin{array}{cc} \sum_{k=1}^{k_{1}+k_{2}}{\left(\omega_{i}\right)}_{j} & -\omega_{i}^{T}\\ -\omega_{i} & diag(\omega_{i}) \end{array}\right]$$

where *diag*(⋅) is the diagonalization operation. Then Equation (10) can be reduced to:

(12) $$\arg\underset{Y_{i}}{\min}\;tr\left(\lambda_{i}Y_{i}L_{i}Y_{i}^{T}\right)$$

where *tr*(⋅) is the trace operator. Furthermore, all the $Y_{i}$ are taken into account, and then

(13) $$\begin{array}{l} \arg\underset{Y}{\min}\sum_{i=1}^{N}tr\left(\lambda_{i}Y_{i}L_{i}Y_{i}^{T}\right)\\ =\arg\underset{\alpha }{\min}[tr(AGA^{T})\alpha^{2}+tr(AGB^{T}+BGA^{T})\alpha +tr(BGB^{T})] \end{array}$$

where $G=\sum_{i=1}^{N}(\lambda_{i}X_{i}^{e}L_{i}X_{i}^{eT})$ and weight coefficient α can be uniquely determined. Hence, the spectral-spatial relationship is described and hidden information is explored. It can be seen from Equation (13) that the weight coefficient training is actually an additional information extraction operation. In other words, the most discriminative features in the spectral and spatial features are extracted by introducing α in this procedure.

**Figure 1:**
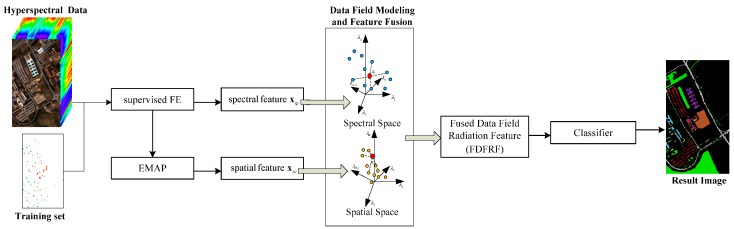
The implementation scheme of the proposed algorithm.

The implementation scheme of the proposed algorithm for hyperspectral imagery classification is shown in Figure 1. As shown the data field modeling operation is implemented in both the spectral space and image spatial domain. Based on the prior information provided by the training set, which consists of spectral information, local spatial information, and label information, the spectral features can be obtained by supervised FE techniques. The spatial structural features can be extracted by the spatial feature extraction algorithms, such as EMP, EAP, and EMAP. The data field modeling operation is carried out in the two spaces, and then the DFRF is built. The feature fusion with local information is then performed. This process fuses the spectral and spatial features into an FDFRF, and learns the fusing weight coefficient. For an unlabeled test pixel, we extract the spectral and spatial features. Then, the extracted features are fused into an FDFRF based on data field modeling. Finally, the classification is implemented by classifiers.

## 3. Experiments and Results

Two standard datasets, the Reflective Optics Systems Imaging Spectrometer (ROSIS-03) University of Pavia dataset and the Airborne Visible Infrared Imaging Spectrometer (AVIRIS) Indian Pines dataset, which are frequently used in research, were used in this study.

The first test dataset is a hyperspectral dataset collected from the University of Pavia, Italy, by the ROSIS-03 airborne instrument. In this dataset, nine classes of interest were considered in the image scene. This dataset, which is composed of 103 bands of 610 × 340 pixels, provides a high spatial resolution of 1.3 m/pixel. The training and test sets were composed of 3909 and 42,788 samples, respectively. The number of training and test samples is shown in Table 1.

The Indian Pines dataset is a standard test dataset acquired in 1992 using the AVIRIS sensor. The data consists of 145 × 145 pixels with a medium spatial resolution of about 20 m/pixel. In this test case, the spectral channels in the atmosphere absorption bands were removed, so 200 data channels were used. Sixteen classes of interest were considered. For this dataset, a total of 695 pixels and 9671 pixels were used to make up the training and test sets, respectively. The number of available test and training samples is displayed in Table 2.

**Figure 2:**
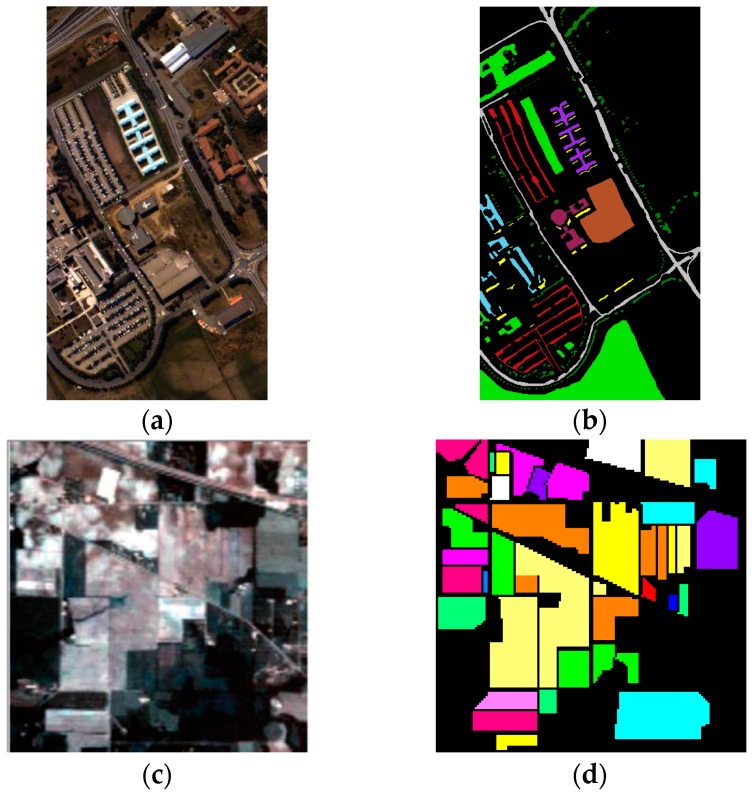
False color representation and corresponding ground truth of (**a**,**b**) ROSIS-03 University of Pavia dataset, 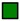 Trees, 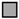 Asphalt, 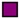 Bitumen, 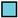 Gravel, 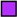 Metal sheets, 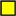 Shadows, 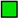 Meadows, 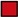 Bricks, 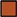 Bare soil; (**c**,**d**) AVIRIS Indian Pines dataset, 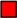 Alfalfa, 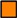 Corn-notil, 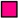 Corn-mintill, 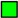 Corn, 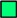 Grass-pasture, 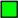 Grass-trees, 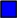 Grass-pasture-mowed, 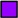 Hay-windrowed, 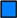 Oats, 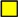 Soybean-notill, 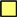 Soybean-mintill, 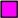 Soybean-clean, 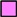 Wheat, 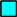 Woods, 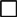 Bldg-grass-tree-drives, 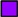 Stone-steel-towers.

The details of the training and test sets of the two datasets are given in References [27,36]. To maintain consistency with previous results, we used the same size training and test sets adopted by other state-of-the-art approaches. We also adopted samples with precisely the same spatial locations as in the previous studies. Each method was executed only once because the samples that we used were identical to those used in the previous studies. False color images of the two datasets are presented in Figure 2.

### 3.1. Experimental Setup

In all the experimental datasets, the spectral-spatial classification method $\eta_{n}$ which was proposed in Reference [27], known as AUTOMATIC, was employed for comparison. The FE approach used is denoted by *n*. Here, the HSI data were first transformed by the FE approach. The spectral feature $x_{\varphi }$ was the output of this step. Next, the spatial feature $x_{\omega }$ was obtained by EMAP and the FE approach. Finally, $x_{\varphi }$ and $x_{\omega }$ were stacked together for classification. DAFE and DBFE were employed for supervised FE. DAFE is often applied to dimension reduction and feature extraction in a pattern recognition field. The class centers and covariance matrix of each class are calculated by training samples in DAFE. As a parametric method, DAFE achieves a satisfactory performance if the data approximately follow a normal distribution. DBFE extracts both discriminately informative and redundant features from the decision boundary. Using the decision boundary feature matrix, the decision boundary is described and features are extracted. For example, $\eta_{DA}$ denotes that the raw data were first transformed by DAFE. Then the EMAP was performed on the baseline images obtained by DAFE. Finally, the spectral features extracted by DAFE and the spatial features obtained by EMAP were stacked together.

It should be emphasized that the features containing more than 99% of cumulative eigenvalues were selected when DAFE and DBFE were employed in the following experiments. The classification results obtained by using the spectral information were reported only for comparison. We use DA and DB to indicate the spectral information extracted by DAFE and DBFE, respectively. The EMAP methods were also employed to demonstrate the superiority of the proposed algorithm. The DA*_p_* and DB*_p_* denote the EMAPs that were generated based on the features extracted by DAFE and DBFE, respectively. The EMAP-based classification methods proposed in References [25,26], which were respectively denoted by GA and SUnSAL, were employed. The recent state-of-the-art spectral-spatial classification approaches, including MH [29] and LBP [30], were used for comparisons. For the MH approach, the hypotheses for prediction were generated using the manually selected spectral-band partitions as suggested in [29]. In the LBP method, the criterion of linear prediction error (LPE) [37] was used for spectral band selection, and LBP features were extracted on these selected bands. Then, the LBP features and selected spectral bands were fused at the feature level, and processed by the classifier. To make our methods fully comparable with the reference techniques, the thresholds and values used for this experimental setup were selected from References [15,27].

The term ${\text{ℱ}}_{n}$ signifies our proposed method. The FE approach is denoted by *n*. The spectral feature $x_{\varphi }$ and spatial feature $x_{\omega }$ were fused into FDFRF in our proposed method. In the experiments, we set k=5, i.e., five NNs in each class were considered in the data field modeling. The features extracted by all the methods were analyzed by an RF classifier. In all the experiments, the number of trees was set to 200, as suggested in References [15,35,36], in order to achieve a trade-off between the classification performance and time cost for the learning phase. The method performances were evaluated by three measurements: the overall accuracy (OA), the average accuracy (AA), and the Kappa coefficient (κ). However, in order to avoid unnecessary redundancy in the following, the experimental results and comparison will only be analyzed based on OA.

### 3.2. Results

**Figure 3:**
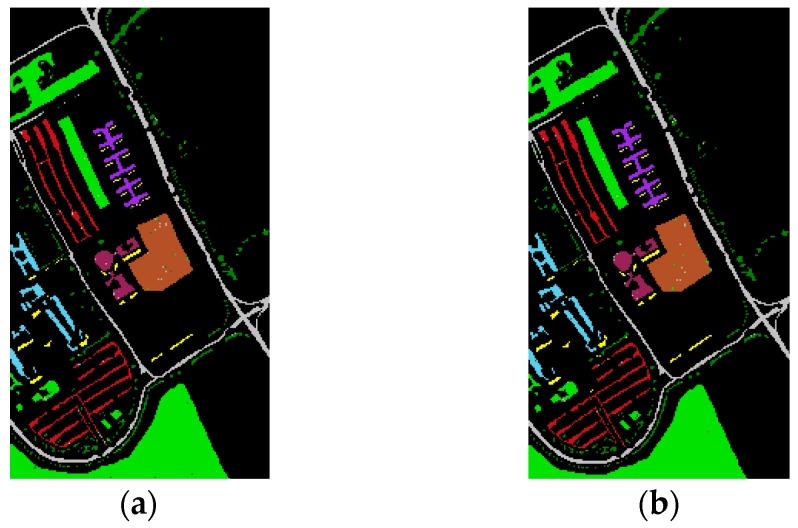
ROSIS-03 University of Pavia dataset. The classification maps of the proposed method obtain by the random forest classifier. (**a**) ${\text{ℱ}}_{\mathrm{DA}}$; (**b**) ${\text{ℱ}}_{\mathrm{DB}}$. 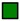 Trees, 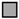 Asphalt, 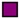 Bitumen, 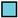 Gravel, 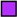 Metal sheets, 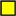 Shadows, 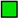 Meadows, 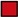 Bricks, 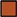 Bare soil.

**Figure 4:**
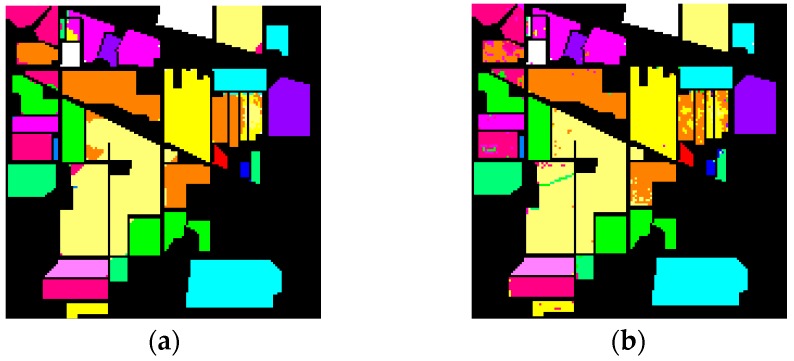
AVIRIS Indian Pines dataset. The classification maps of the proposed method obtain by the random forest classifier, (**a**) ${\text{ℱ}}_{\mathrm{DA}}$; (**b**) ${\text{ℱ}}_{\mathrm{DB}}$. 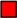 Alfalfa, 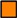 Corn-notil, 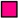 Corn-mintill, 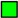 Corn, 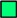 Grass-pasture, 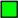 Grass-trees, 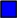 Grass-pasture-mowed, 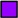 Hay-windrowed, 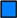 Oats, 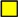 Soybean-notill, 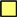 Soybean-mintill, 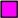 Soybean-clean, 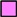 Wheat, 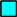 Woods, 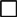 Bldg-grass-tree-drives, 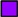 Stone-steel-towers.

As shown in Table 3 and Table 4, the results of our experiments with the two datasets show that feature fusion based on the data field theory can improve classification accuracy compared to the reference methods. The classification results acquired by the proposed method on the two datasets by the proposed method are shown in detail in Figure 3 and Figure 4.

For the University of Pavia dataset, the data field feature fusion resulted in significantly improved classification accuracy. As can be observed from Table 3, ${\text{ℱ}}_{\mathrm{DB}}$ outperformed the other methods with an OA of 99.4%. ${\text{ℱ}}_{\mathrm{DA}}$ achieved 19%, 4.1%, and 13.4% improvement in OA over DA, DA***_p_*** and $\eta_{DA}$, respectively. Compared with the corresponding reference DB, DB***_p_*** and $\eta_{\mathrm{DB}}$ methods, ${\text{ℱ}}_{\mathrm{DB}}$ improved the OA by 20.5%, 3.4%, and 2.6%, respectively. It is also important to emphasize that $\eta_{\mathrm{DB}}$ exhibited excellent classification performances with an OA of 96.8%. In comparison, ${\text{ℱ}}_{\mathrm{DA}}$ and ${\text{ℱ}}_{\mathrm{DB}}$ achieved small improvements in OA of 2.1% and 2.6%, respectively. Although the improvements in classification accuracy are not remarkable in the manner of OA, more than 65.6% and 81.2% of test samples misclassified by $\eta_{DB}$ were corrected by ${\text{ℱ}}_{\mathrm{DA}}$ and ${\text{ℱ}}_{\mathrm{DB}}$, respectively. We can therefore conclude that the proposed method effectively improved the classification performance.

Compared with the results reported in Table 3, it is easy to deduce that DBFE outperforms DAFE. The primary reason may be that DAFE is not full rank, so that some discriminative spectral information was lost. It should be noted that the classification performances of AUTOMATIC, which stacked the spectral and spatial features together, were affected by different FE approaches. The OA resulting from $\eta_{\mathrm{DB}}$ is 11.3% more than that of $\eta_{\mathrm{DA}}$. Compared to the EMAP approaches, AUTOMATIC improved the classification accuracy when DBFE was employed. However, AUTOMATIC classification decreased when DAFE was performed. The proposed method is much more robust with respect to the choice of the FE technique. Classification results always remained at a high level when different FE approaches were used. This is because our method further fused the extracted spectral and spatial features. As a result, the useful information that lies in the spectral-spatial relationship and can contribute to the classification was included. 

Compared with the employed state-of-the-art HSI classification methods, the proposed method additionally achieved competitive classification performance in this test case. ${\text{ℱ}}_{DB}$ achieved the best classification results in terms of OA, AA and the κ value. As can be observed from the classfication results, ${\text{ℱ}}_{\mathrm{DB}}$ achieved approximately 3.3%, 1.3%, 0.6% and 0.2% improvements in OA over GA, SUnSAL, LBP and MH, respectively. Though the OA improvements are seemingly very small, almost 84.6%, 68.4%, 50% and 25% of misclassified samples in these methods were corrected, respectively. Moreover, ${\text{ℱ}}_{\mathrm{DA}}$ also produced a satisfactory classification performance with an OA of 98.9%. Although the MH approach reported a higher classification accuracy with an OA of 99.2%, ${\text{ℱ}}_{\mathrm{DA}}$ is competitive because it perfomed better than all the other reference methods.

In contrast to the University of Pavia dataset, the low spatial resolution, which leads to more mixed pixels, makes the classification task more complex in the Indian Pines dataset. For this test case, the HSI classification results, obtained by further feature fusion, were generally better than the corresponding compared methods. For example, ${\text{ℱ}}_{\mathrm{DA}}$ achieved 33.3%, 5.7%, and 3.5% improvements in OA over DA, DA***_p_***, and $\eta_{\mathrm{DA}}$, respectively. ${\text{ℱ}}_{\mathrm{DB}}$ improved the OA of DB, DB***_p_***, and $\eta_{\mathrm{DB}}$ by 31.9%, 5.4%, and 11.9%, respectively. The best accuracies were obtained by using ${\text{ℱ}}_{\mathrm{DA}}$ which achieved an OA of 96.8%. It should be noted that reference methods exhibited acceptable performances in terms of classification accuracies. In contrast, the ${\text{ℱ}}_{\mathrm{DA}}$ achieved the best performances in 11 classes and ${\text{ℱ}}_{\mathrm{DB}}$ performed better than all the reference methods in 11 classes. As the results represented in Table 4 show, DAFE performs better than DBFE in terms of OA, AA, and the Kappa coefficient. A possible reason may be that the presence of the pixels with mixed spectra leads to the features number extracted by DBFE being insufficient to discriminate the samples in different classes.

In the Indian Pines dataset, the results also indicate that the AUTOMATIC approach is affected by different FE methods. Our method avoided this problem by using data field modeling and further feature fusion. As can be observed from the classification results reported in Table 4, the state-of-the-art spectral-spatial methods improved the classification more significantly than the spectral-based methods DA and DB in this test case. This may be because the spectral information is less dominant in this test case and introducing spatial information effectively contributes to the classification problem. As with the Pavia University dataset, our method obtained competitive results for this dataset in comparison to the other state-of-the-art methods. The best classification result was obtained by ${\text{ℱ}}_{\mathrm{DA}}$ with an OA of 96.8%, and the missclassified rates decreased approximately 48.4%, 46.6%, 52.2% and 25.6% compared to GA, SUnSAL, LBP and MH, respectively. Moreover, ${\text{ℱ}}_{\mathrm{DB}}$ also performed competitively with better classification accuracies than the other reference methods, except for MH.

As Equation (5) shows, the feature number (i.e., the dimensionality of the FDFRFs) in our method is determined by the number of the classes and NNs used in the data field modeling. The feature numbers of our method were 45 and 80 using the Pavia University dataset and Indian Pines dataset, respectively. The proposed method can be seen as an advancement of the AUTOMATIC approach. Accordingly, the feature numbers of the proposed method and AUTOMATIC are listed in Table 5. It can be seen from Table 5 that the proposed method achieved better classification results with acceptable feature numbers. Compared to the EMAP reference methods, our methods effectively reduced the feature numbers and improved classification accuracy. Moreover, ${\text{ℱ}}_{\mathrm{DB}}$ (consisting of 45 features) performed better than $\eta_{\mathrm{DB}}$, which consisted of 59 features in the Pavia University dataset. In the Indian Pines dataset, the proposed method also showed superior classification performance over AUTOMATIC approaches with an acceptable feature number.

Finally, we compare the computational complexity of the classification methods. As an example, the processing times (in seconds) of the methods with the Indian Pines dataset are shown in Table 6. All experiments were implemented using MATLAB on an Intel Core i5 CPU with 3.2 GHz and 4 GB of RAM. As can be seen in Table 6, the DAFE-based methods have an obvious advantage in computational time compared to DBFE-based approaches because DAFE is faster than DBFE. The computational costs of data field-based methods are higher than those of the corresponding AUTOMATIC approaches owing to the burden of building FDFRFs. Compared to the other methods, our method achieved superior classification performances at the cost of greater computational complexity and time consumption. However, the speed of our method could be improved by using time-efficient feature extraction approaches and parallel computing techniques.

### 3.3. Parameters

In this section, two important parameters used in the presented algorithm are discussed. First, the radiation factors used in the radiation function are analyzed, and an adaptive method for determining the radiation factor is put forward. Secondly, the relationship between the algorithm performance and k, the number of NNs used in data field modeling, is discussed.

As shown in Equation (1), the radiation intensity is jointly determined by the distance measurement d and the radiation factor. Radiation factors determine the character of the radiation effects in data fields or, for simplicity, the range of the data radiation domain. The distance measurement can lose meaning when ρ is extremely small or large. The data interact strongly when ρ is very small, whereas, the interactions between data are negligible if ρ is very large. Additionally, as before, we use different radiation factors in different spaces and classes when calculating radiation intensities. In this study, the values of the radiation factors were determined by the training samples. For a given training sample $\left(x_{i},l\right)$, the training set can be divided into two parts, as mentioned in Section 2. The vector mean value of the Same Class Subset spectral features is denoted by ${\bar{x}}_{i,\varphi }$, which can be considered as the center of the class l in the spectral feature space. It is desirable for the training samples in the same class and different classes to have as strong and weak radiations as possible, respectively, i.e.,:

(14) $$\begin{array}{l} \rho_{\varphi }^{l^{*}}=\arg\underset{\rho }{\max}(e^{-\rho d_{+,\varphi }^{2}}-e^{-\rho d_{-,\varphi }^{2}})\\ \rho_{\varphi }^{l^{*}}=2(\ln d_{+,\varphi }-\ln d_{-,\varphi })/\left(d_{+,\varphi }^{2}-d_{-,\varphi }^{2}\right) \end{array}$$

where $d_{+,\varphi }$ is the mean value of the distances between ${\bar{x}}_{i,\varphi }$ and the samples in the Same Class Subset, and $d_{-,\varphi }$ is the mean value of the distances from ${\bar{x}}_{i,\varphi }$ to the samples in the Different Class Subset. Therefore, $\rho_{\varphi }^{l}$ (i.e., the radiation factor of $\left(x_{i},l\right)$ in the spectral domain data field) can be adaptively determined by the training samples. The radiation factor of $\left(x_{i},l\right)$ in the spatial domain data field, which is denoted by $\rho_{\omega }^{l}$, can be determined in the same way.

**Figure 5:**
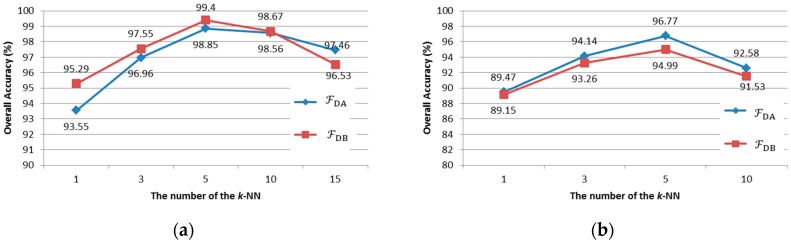
Overall accuracy with different number of NNs. (**a**) Pavia University dataset; (**b**) Indian Pines dataset.

In our proposed method, the number of NNs *k* is the most important parameter in determining the data field modeling accuracy and classification performance. The influence of *k* on the algorithm performance, measured by OA, can be observed in Figure 5. Note that OA increases with *k*. However, the classification performance decreases when k>5 and k>10 in the Indian Pines dataset and Pavia University dataset, respectively. This is because a large *k* may lead to a higher dimension of FDFRF, which may cause the Hughes phenomenon. Moreover, a large *k* also brings a higher computation cost. Based on our experimental results, it is reasonable to set *k* = 5, which avoids the Hughes phenomenon and achieves a good trade-off between the classification performance and computation cost.

### 3.4. Experiments Using Reduced Training Samples

**Figure 6:**
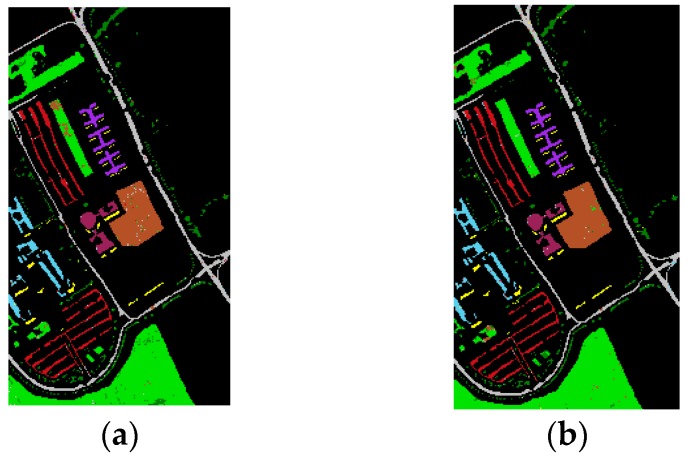
ROSIS-03 Pavia University dataset. The classification maps of the proposed method with 30 training pixels per class. (**a**) ${\text{ℱ}}_{\mathrm{DA}}$; (**b**) ${\text{ℱ}}_{DB}$. 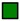 Trees, 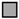 Asphalt, 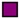 Bitumen, 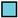 Gravel, 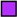 Metal sheets, 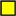 Shadows, 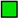 Meadows, 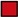 Bricks, 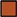 Bare soil.

As can be observed in Table 1 and Table 2, a large training set with 3909 training samples was used in the University of Pavia test case and a relatively small training set was employed in the Indian Pines dataset with 15 or 50 training pixels per class. In order to further validate the classification performance using a small training sample size, an additional experiment was performed using the Pavia University dataset with a reduced number of training samples. In this experiment, 30 training samples per class were randomly selected from the provided 3909 training samples to form the small training sample set. Table 6 reports the classification OA, AA, κ value, and individual class accuracies achieved by different approaches. The classification maps acquired by our proposed method using the small training sample size are shown in Figure 6. As can be observed in Table 7, ${\text{ℱ}}_{\mathrm{DB}}$ and LBP achieved the best classification performance in terms of OA, with an OA of approximately 96.6%. However, LBP performed better in terms of the AA and κ value and obtained the smallest degradation in OA. The reason might be because the LBP approach can extract the detailed local image characteristics, such as corners, edges and knots. Hence, it is more efficient and robust in describing spatial features than EMAP-based methods, particularly in the small training sample size case. ${\text{ℱ}}_{\mathrm{DA}}$ also demonstrated a competitive performance under the small training sample size. Compared with all the reference methods except LBP and ${\text{ℱ}}_{\mathrm{DB}}$, ${\text{ℱ}}_{\mathrm{DA}}$ obtained higher classification accuracies. Therefore, it can be concluded that our proposed technique can achieve satisfactory classification results with limited training data.

## 4. Discussion

The experimental results demonstrate that feature fusion can further promote accurate classification performance. Compared to the reference methods, which simply fused the extracted features via vector stacking, the proposed method further fused the spectral and spatial information through the introduction of data field theory. A relationship between the spectral and spatial features was built and previously hidden information was explored. It can be concluded from our results that our method fused the spectral and spatial features in a reasonable and effective way. Furthermore, the proposed method is robust to the FE approaches, which is also desirable.

Two standard hyperspectral data sets were employed to measure the efficacy of our proposed method. The two test cases represent two typical types of classification problems. The Pavia University dataset covers an urban area with both high spectral and spatial resolution. It is a typical urban classification problem. The Indian Pines dataset, with relatively low spatial resolution, represents agriculture land-cover problems. The experimental results obtained on both datasets demonstrate that our proposed method is generally applicable to different classification problems.

A subject for future investigation is the optimization of data field modeling based on an imaging mechanism. The fusing model used in this paper is a linear weighted addition model. A more reasonable and effective model will be studied in future research. Another subject that deserves further research is the adaptive selection of the number of NNs used in the data field modeling.

## 5. Conclusions

In this study, a feature fusion method based on data field theory was proposed to carry out the supervised classification of HSI. As a mathematical realization of field theory concepts in physics, data field theory was employed to establish data field modeling in HSI. Both the spectral features and spatial space were considered data fields. The fusion weight coefficient was trained based on the data field modeling. Thus, a relationship between the spectral and spatial feature was constructed, and the two features were fused into a discriminative FDFRF. The weight coefficient training procedure was a further feature extraction process. The relationship between the spectral and spatial information was explored and the method was shown to achieve improved classification performance.

## Figures and Tables

**Table 1:** University of Pavia dataset: number of training and test samples.

Class		Number of Samples	
Labels	Name	Training	Test
1	Asphalt	548	6631
2	Meadow	540	18,649
3	Gravel	392	2099
4	Trees	524	3064
5	Metal Sheets	256	1345
6	Bare Soil	532	5029
7	Bitumen	375	1330
8	Bricks	514	3682
9	Shadows	231	947

**Table 2:** Indian Pines dataset: number of training and test samples.

Class		Number of Samples	
Labels	Name	Training	Test
1	Alfalfa	15	39
2	Corn-notil	50	1384
3	Corn-mintill	50	784
4	Corn	50	184
5	Grass-pasture	50	447
6	Grass-trees	50	697
7	Grass-pasture-mowed	15	11
8	Hay-windrowed	50	439
9	Oats	15	5
10	Soybean-notill	50	918
11	Soybean-mintill	50	2418
12	Soybean-clean	50	564
13	Wheat	50	162
14	Woods	50	1244
15	Bldg-grass-tree-drives	50	330
16	Stone-steel-towers	50	45

**Table 3:** University of Pavia dataset: OA, AA and κ value of the classification results. The best classification accuracies are marked in bold.

Class Labels	DA	DB	DA*_p_*	DB*_p_*	$\eta_{\mathrm{DA}}$	$\eta_{\mathrm{DB}}$	GA	SUnSAL	LBP	MH	${\text{ℱ}}_{\mathrm{DA}}$	${\text{ℱ}}_{\mathrm{DB}}$
1	82.8	84.9	98.0	98.1	98.3	96.7	95.5	97.5	96.8	99.1	99.4	99.5
2	72.1	66.9	92.6	94.4	69.0	95.8	95.7	97.8	99.3	99.4	99.5	99.7
3	71.9	67.5	81.0	98.0	91.6	87.0	76.8	99.1	97.2	97.8	99.5	99.5
4	92.8	93.9	97.8	87.3	99.5	99.3	96.7	97.7	98.9	99.3	95.7	98.9
5	100	99.9	99.8	99.6	100	99.8	99.6	100	100	100	100	100
6	89.1	93.8	98.6	100	99.7	99.9	99.5	99.4	100	99.2	98.5	99.2
7	83.8	85.5	100	100	99.7	99.9	100	99.2	99.6	99.5	97.2	98.9
8	82.8	87.5	96.1	98.1	99.4	99.4	99.5	97.0	98.0	98.9	98.0	98.9
9	98.0	98.2	94.5	97.1	92.4	91.8	97.3	100	100	100	96.8	100
OA(%)	79.9	78.9	94.8	96.0	85.5	96.8	96.1	98.1	98.8	99.2	98.9	99.4
AA(%)	85.9	86.5	95.1	97.0	94.4	96.7	96.0	98.6	98.9	99.2	98.3	99.5
κ	0.75	0.74	0.93	0.95	0.82	0.96	0.95	0.97	0.97	0.98	0.98	0.99

**Table 4:** Indian Pines dataset: OA, AA and κ value of the classification results. The best classification accuracies are marked in bold.

Class Labels	DA	DB	DA*_p_*	DB*_p_*	$\eta_{\mathrm{DA}}$	$\eta_{\mathrm{DB}}$	GA	SUnSAL	LBP	MH	${\text{ℱ}}_{\mathrm{DA}}$	${\text{ℱ}}_{\mathrm{DB}}$
1	53.9	48.7	97.4	97.4	94.8	97.4	97.4	100	100	100	100	100
2	53.0	49.1	82.7	79.9	88.5	73.7	82.7	83.7	91.4	95.7	95.2	88.0
3	52.2	48.0	96.0	96.4	95.1	90.4	97.2	94.1	97.1	92.2	99.1	94.0
4	78.3	70.1	92.9	88.5	98.9	94.0	100	92.9	100	99.5	97.9	99.5
5	84.1	79.6	93.7	93.5	94.6	93.2	93.1	93.3	98.4	93.5	98.8	95.5
6	88.4	89.2	96.1	99.0	97.1	98.5	99.4	99.7	98.5	99.6	99.6	100
7	100	81.8	100	100	100	100	100	100	100	100	100	92.3
8	98.2	98.6	99.7	99.3	98.6	99.0	99.8	100	100	97.7	100	100
9	40.0	20.0	100	100	100	80.0	100	100	100	100	100	100
10	60.5	62.3	91.6	87.2	86.6	77.8	89.1	92.3	90.1	95.6	91.7	91.9
11	39.0	42.7	85.1	82.0	91.3	70.3	94.3	92.4	86.8	93.1	94.2	93.4
12	66.0	68.1	87.7	84.9	89.7	77.6	90.8	98.5	83.7	94.1	98.9	98.2
13	97.5	98.8	99.3	100	99.3	100	98.2	100	100	99.4	99.3	99.4
14	84.8	85.1	99.3	99.6	99.4	93.1	99.6	100	99.9	98.6	100	99.8
15	82.1	70.0	99.0	98.7	99.3	98.4	97.6	97.4	100	99.4	100	99.7
16	97.8	93.3	100	100	100	100	100	97.7	100	97.8	100	97.8
OA(%)	63.5	63.1	91.1	89.6	93.3	83.1	93.8	94.0	93.3	95.7	96.8	95.0
AA(%)	73.5	69.1	95.1	94.2	95.9	90.3	96.2	96.4	96.6	97.3	98.4	96.8
κ	59.0	58.5	0.90	0.88	0.92	0.81	0.93	0.92	92.8	0.95	0.96	0.93

**Table 5:** The number of features used for classification in the proposed method and the corresponding AUTOMATIC approaches in the two test cases.

Algorithms	Features	
	University of Pavia	Indian Pines
DA	6	13
DB	29	16
DA***_p_***	306	663
DB***_p_***	1479	816
$\eta_{\mathrm{DA}}$	14	26
$\eta_{\mathrm{DB}}$	59	59
${\text{ℱ}}_{DA}$	45	80
${\text{ℱ}}_{DB}$	45	80

**Table 6:** Processing time (in seconds) for Indian Pines dataset with 695 training samples and 9671 test samples.

Algorithms	Time (s)
DA	2
DB	39
DA*_p_*	13
DB*_p_*	45
$\eta_{\mathrm{DA}}$	19
$\eta_{\mathrm{DB}}$	133
GA	14
SUnSAL	19
LBP	15
MH	253
${\text{ℱ}}_{\mathrm{DA}}$	67
${\text{ℱ}}_{\mathrm{DB}}$	512

**Table 7:** Accuracies in percentage for classification of the Pavia image with 30 training pixels per class. The best results in terms of accuracy are marked in bold.

Class Labels	DA	DB	DA*_p_*	DB*_p_*	$\eta_{\mathrm{DA}}$	$\eta_{\mathrm{DB}}$	GA	SUnSAL	LBP	MH	${\text{ℱ}}_{\mathrm{DA}}$	${\text{ℱ}}_{\mathrm{DB}}$
1	74.0	76.4	91.9	89.1	89.8	91.3	88.7	92.8	91.3	93.6	96.8	96.9
2	68.9	70.8	81.6	87.5	69.7	90.8	90.7	88.4	98.4	95.4	94.4	96.3
3	70.9	67.1	72.3	63.2	96.4	98.9	80.5	96.2	97.0	96.5	97.2	95.6
4	90.6	89.1	87.1	87.6	88.1	95.6	93.4	93.9	96.5	92.9	91.9	94.1
5	99.9	100	100	99.9	100	99.9	99.3	99.7	100	100	100	100
6	76.3	82.1	87.0	90.5	87.8	94.6	92.6	88.8	96.3	95.3	96.3	98.4
7	87.8	88.6	86.1	98.6	95.5	99.6	94.9	99.7	98.7	98.3	97.9	95.8
8	73.8	82.1	71.9	87.0	70.6	92.0	92.5	94.5	94.5	95.2	96.1	96.6
9	99.9	99.7	99.7	99.4	99.8	99.9	96.5	98.4	100	97.6	95.3	97.1
OA(%)	74.9	77.2	84.0	87.9	80.0	92.9	91.0	90.9	96.6	95.2	95.4	96.6
AA(%)	82.5	84.0	86.4	89.2	80.6	95.8	92.1	93.6	97.0	96.1	96.2	96.7
***K***	0.68.	0.71	0.79	0.84	0.75	0.91	0.88	0.88	0.96	0.93	0.94	0.96
